# The Italian Trend of Contact Allergy to 2‐Hydroxyethyl Methacrylate: Is the Current European Legislation Working?

**DOI:** 10.1111/cod.14800

**Published:** 2025-05-21

**Authors:** Elena Sofia Caroppo, Gabriele Casciola, Katharina Hansel, Alba Guglielmo, Silvia Mariel Ferrucci, Fabrizio Guarneri, Maria Michela Lauriola, Maddalena Napolitano, Cataldo Patruno, Paolo Romita, Donatella Schena, Ilaria Trave, Leonardo Bianchi, Luca Stingeni, 2‐HEMA Study Group, Elisa Marzola, Francesco Bellinato, Paolo Calzari, Francesca di Vico, Rosella Gallo, Roberta Giuffrida, Benedetta Tirone, Marta Tramontana

**Affiliations:** ^1^ Dermatology Section, Department of Medicine and Surgery University of Perugia Perugia Italy; ^2^ Section of Dermatology and Infectious Diseases, Department of Medical Sciences University of Ferrara Ferrara Italy; ^3^ Dermatology Unit Fondazione IRCCS Ca' Granda Ospedale Maggiore Policlinico Milan Italy; ^4^ Section of Dermatology, Department of Clinical and Experimental Medicine University of Messina Messina Italy; ^5^ Istituti Ospedalieri Bergamaschi, Policlinico San Marco Dermatology Unit Italy; ^6^ Section of Dermatology, Department of Clinical Medicine and Surgery University of Naples Federico II Naples Italy; ^7^ Department of Medicine and Health Sciences Vincenzo Tiberio University of Molise Campobasso Italy; ^8^ Section of Dermatology, Department of Precision and Regenerative Medicine and Jonian Area University Aldo Moro of Bari Bari Italy; ^9^ Section of Dermatology and Venereology, Department of Medicine University of Verona Verona Italy; ^10^ Section of Dermatology, Department of Health Sciences University of Genoa, IRCCS, Ospedale Policlinico San Martino Genoa Italy

**Keywords:** 2‐hydroxyethyl methacrylate, acrylates, contact allergy, European legislation, methacrylates, patch test

## Abstract

**Background:**

(Meth)acrylates are well‐known causes of allergic contact dermatitis, and nail products are the major source of exposure, especially in a non‐occupational setting. In 2020, the European legislation restricted 2‐hydroxyethyl methacrylate (2‐HEMA) only in nail cosmetics to professional use.

**Objective:**

To investigate the Italian prevalence trend of positive patch test reactions to 2‐HEMA, the best marker for delayed hypersensitivity to (meth)acrylates.

**Methods:**

A retrospective descriptive study on consecutive patients patch tested with the Italian baseline series containing 2‐HEMA was performed in 8 Italian Dermatology Clinics. Demographics, patch test results, exposure settings and sources were analysed.

**Results:**

Amongst 7133 patch tested patients, 147 (2.1%) resulted positive to 2‐HEMA with an increasing trend from 2019 (1.6%) to 2023 (2.7%). The median age of sensitised female patients with a relevant positive patch test reaction to 2‐HEMA was significantly decreasing over time (*p* = 0.004). Non‐occupational allergic contact dermatitis was documented in 68.7%; artificial nails were the leading source of exposure in both occupational (75.0%) and non‐occupational (72.2%) settings.

**Conclusion:**

The prevalence of contact allergy to 2‐HEMA in Italy shows a progressively increasing trend over time. Currently, the European legislation does not appear to be effective in preventing or reducing this phenomenon.

## Graphical abstract

From 2019 to 2023, 7133 Italian consecutive patients underwent patch test with 2‐HEMA. 147 (2.1%) resulted positive (78.2% relevant), with an increasing trend over years and a decreasing female median age. Artificial nails were the leading source of exposure in both occupational (75.0%) and non‐occupational (72.2%) settings.

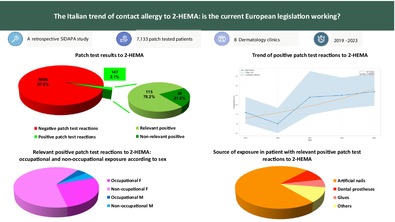

## 1 Introduction

Acrylates and methacrylates, from now on referred to as (meth)acrylates, are thermoplastic resins derived from the polymerisation of reactive monomers of acrylic or methacrylic acid [1]. The monomers polymerise, both spontaneously and under the effect of heat and ultraviolet (UV) light exposure, to form products widely used in a large variety of products as dental materials, medical devices, adhesive tapes in biomedical applications, nail products, diapers, paints and coatings, and electronic devices [2, 3]. Acrylic and methacrylic monomers have a high sensitising potential and represent an important and increasing cause of occupational and non‐occupational allergic contact dermatitis (ACD) [1], with a prevalence of contact allergy ranging from 1.6% to 3.6% in Europe, and above 3% in North America [1]. In 2018, we documented a prevalence of positive patch test reactions to 2‐hydroxyethyl methacrylate (2‐HEMA) in Italian consecutively patch tested patients of 1.5%, mainly in females where this prevalence reached 2.1% [4]. The large female prevalence is reported in other studies and confirms the leading role of nail cosmetics in ACD to (meth)acrylates [1, 5].

In the past, ACD caused by (meth)acrylates was considered to be a primarily occupational disease, affecting mainly dentists, dental and prosthesis technicians, workers in fibre‐glass and graphic printing industries, as well as painting and coating workers [3, 6]. In the past 20 years, the increasing use of nail cosmetics (acrylate nails, gel nails, nail wraps, powder nails, press‐on nails and long‐lasting nail polish) has caused an outbreak of ACD by (meth)acrylates, not only in nail technicians but also in artificial nail users [4, 5, 7].

In view of this, in 2020 the European Union (EU) Commission Regulation restricted 2‐HEMA in nail cosmetics to professional use only, with the requirement for the product package to be labelled “For professional use only” and “Can cause an allergic reaction” [8]. From September 2021, “products containing 2‐HEMA without the correct labelling shall not be made available on the EU market” [9]. Despite this, the currently available nail cosmetics often do not comply with the EU regulation [10], with a high risk of ACD to HEMA‐containing nail products [11].

From a diagnostic point of view, 2‐HEMA (2‐hydroxyethyl ester of methacrylic acid) has been increasingly recognised as the marker of contact allergy to (meth)acrylates [4] and 2‐HEMA 2.0% in pet. was first suggested for patch testing as the marker of (meth)acrylates contact allergy by Goon et al. in 2008 [12]. Subsequently, it was added to baseline patch test series by the British Society for Cutaneous Allergy (BSCA) in 2018 and by the European Society of Contact Dermatitis (ESCD) in 2019 [7, 13]. In Italy, it has been added to SIDAPA (Società Italiana di Dermatologia Allergologica, Professionale e Ambientale) baseline series since 2016 [4].

This multicentre Italian retrospective study aimed to assess the 5‐year trend of positive patch test reactions to 2‐HEMA on consecutive patients referred for patch testing, analysing whether the sources of exposure to (meth)acrylates have changed over time.

## 2 Methods

### 2.1 Study Population

This is a retrospective observational study of consecutively patch tested patients enrolled from January 2019 to December 2023 in 8 Dermatology Clinics homogeneously distributed in Northern (Verona and Zingonia Osio Sotto‐Bergamo), Central (Ferrara, Genoa and Perugia) and Southern Italy (Naples, Bari and Messina). The study was approved by the Messina Ethics Committee (protocol no. 135‐22). All patients underwent patch testing with the SIDAPA baseline series containing 2‐HEMA 2.0% in pet.

### 2.2 Patch Testing and Data Collection

Patch tests were performed using Haye's Test Chambers (Haye's Service, Alphen aan den Rijn, the Netherlands) on Soffix tape (Artsana, Grandate, Italy), and allergens were provided by FIRMA Diagent (Florence, Italy) from 2019 to 2021 and by SmartPractice Europe (Greven, Germany) from 2022 to 2023. Patch test readings were performed on day (D)2, D4 and D7 [14]; irritant and doubtful responses were recorded as negative results. The relevance of positive patch test reactions was defined as definite (positive patch test or use test with a skin contactant verified to contain the allergen), probable (allergen verified in skin contactant with a consistent clinical presentation), possible (skin contactant possibly contained the allergen with a consistent clinical presentation) and unknown/absent (skin contactant did not likely contain the allergen or the clinical presentation appeared inconsistent) [15, 16]. We considered the reactions as relevant whenever they did not fall in the last group. The aforementioned data, combined with demographic information (age and sex), sources and settings of exposure, affected body areas, and final diagnoses were subsequently gathered and analysed by year, also considering the recent measures of 2‐HEMA regulation under European legislation.

### 2.3 Statistical Analysis

The Pandas, Matplotlib, SciPy, Numpy and Seaborn libraries, running in Python version 3.12, were used. Categorical data (frequencies and proportion) were presented as percentages, while continuous variables were expressed as median and IQR in graphics. Differences between means of continuous values were assessed using the Mann–Whitney U test, and proportions' confidence intervals and *p* values for differences were calculated with the Wilson method and shown with ribbon plots. Demographic and clinical characteristics of the patch test population were studied by the MOAHLFA index [17], and the relationship between each of its parameters and the result of the patch test to 2‐HEMA was calculated with both crude proportion comparisons and a multivariate logistic regression analysis. To directly compare proportions, the Two Proportion *Z*‐test was used when the patients count with the analysed characteristic was greater than 5; otherwise, the Fisher exact test was used. The presence of trends for non‐normally distributed continuous data was studied with the Spearman rank correlation score. The presence of trends over time in both frequencies and proportions was assessed with a simple linear regression model after applying a logit transform to the y‐axis to normalise the data. The result of these analyses was then graphically displayed with the alleged *p* value, *R* ^2^ and fitted line equation. *p* values were considered significant when *p* < 0.05.

## 3 Results

**FIGURE 1:**
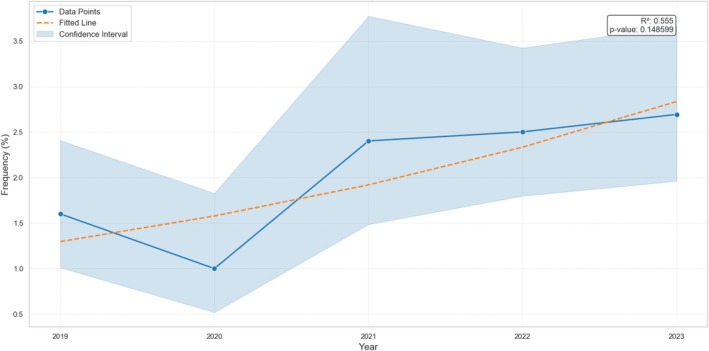
Frequency of positive patch test reactions to 2‐HEMA per year: Non‐significant (but near the significant threshold, *p* = 0.149) upward trend (the confidence interval for the frequency calculated with Wilson method is shown with the ribbon plot).

The positive patch test results to 2‐HEMA from 2019 to 2023, the overall prevalence and, for each year, the proportion of relevant positive reactions, and the distribution by sex with corresponding median ages are shown in Table 1. Over the 5‐year period, a total of 7133 subjects were patch tested. The overall positive reaction prevalence was 2.1% (147 cases), with 78.2% (115 cases) of these deemed relevant. The annual prevalence increased from 1.6% in 2019 to 2.7% in 2023, showing an upward trend, although not statistically significant (*p* = 0.149, Figure 1).

**TABLE 1:** Positive patch test reactions to 2‐HEMA in 7133 consecutive patients.

	2019			2020			2021			2022			2023			Total		
Year	Tested	Positive reactions (%)	Relevant positive reactions (%)	Tested	Positive reactions (%)	Relevant positive reactions (%)	Tested	Positive reactions (%)	Relevant positive reactions (%)	Tested	Positive reactions (%)	Relevant positive reactions (%)	Tested	Positive reactions (%)	Relevant positive reactions (%)	Tested	Positive reactions (%)	Relevant positive reactions (%)
Total	1860	29 (1.6)	22 (75.9)	1091	11 (1.0)	9 (81.8)	999	24 (2.4)	22 (91.7)	1715	43 (2.5)	30 (69.8)	1468	40 (2.7)	32 (80.0)	7133	147 (2.1)	115 (78.2)
Median age	48	53	50	49	40	36	46	31	30	47	37	32	48	36.5	36.5	48	38	37
Females	1274	26 (2.0)	20 (76.9)	725	9 (1.2)	7 (77.8)	715	24 (3.4)	22 (91.7)	1138	40 (3.5)	28 (70.0)	1045	34 (3.3)	27 (79.4)	4897	133 (2.7)	104 (78.2)
Median age	48	53	50	48	36.0	35	46	31.0	30	46	32.5	37.0	48	29.5	28	47	36	33.5
Males	586	3 (0.5)	2 (66.7)	366	2 (0.6)	2 (100)	284	0 (–)	0	577	3 (0.5)	2 (66.7)	423	6 (1.4)	5 (83.3)	2238	14 (0.6)	11 (78.6)
Median age	49	53	44.5	51	45	45.0	47	—	—	49	63	67.5	47	50.5	53	49	51	53
*P* (age)	**0.03**	0.91	0.86	0.40	0.33	0.22	0.51	—	—	**0.002**	0.082	**0.04**	0.48	**0.012**	**0.012**	**0.0002**	**0.007**	**0.004**

*Note:* Statistical significant results are highlighted in bold.

**FIGURE 2:**
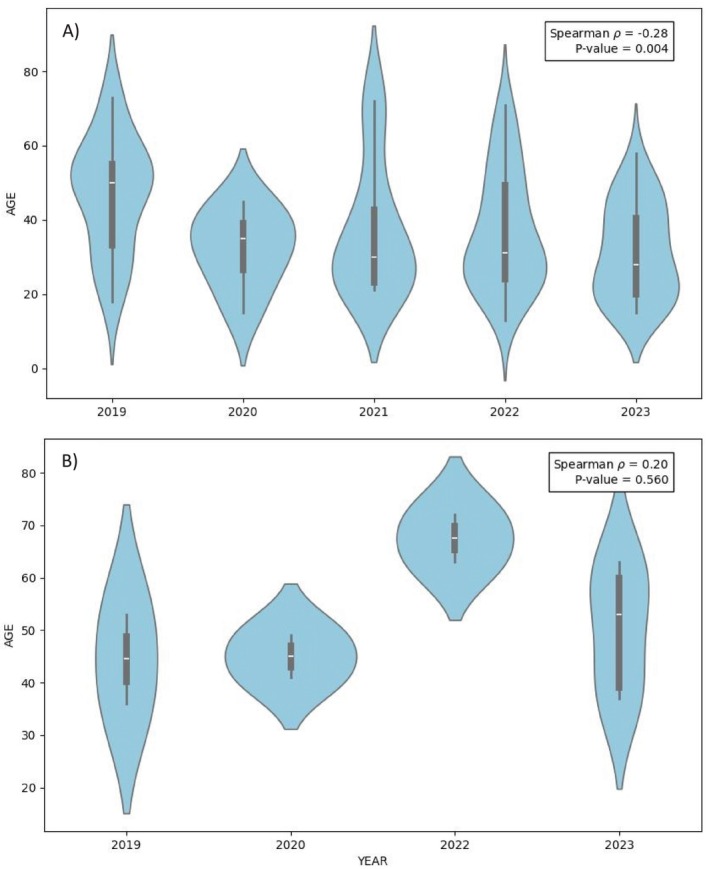
Age of patients with relevant positive patch test reactions to 2‐HEMA, represented by violin plots. Spearman rank correlation tests were performed due to the non‐normality of the age data. (A) For women, a negative correlation over time was found. (B) For men, no correlation was found (in 2021 no male patient with relevant positive reactions to 2‐HEMA).

Sex‐specific data showed some differences: females accounted for 68.7% (4897/7133) of the tested population and exhibited a higher prevalence (2.7%) compared to males (0.6%). Females and males showed a similar rate of relevant positive reactions (78.2% and 78.6%, respectively). The median age of the tested population was consistent across the years, with an overall median of 48 years. However, the overall median age of women with relevant positive patch tests (33.5 years) was significantly lower than that of men (53.0 years) (*p* = 0.004) (Table 1). Furthermore, Spearman rank correlation analysis revealed that over time, the median age of females with relevant positive patch tests decreased significantly (Spearman *r* = −0.28, *p* = 0.004). In contrast, the median age for male patients remained stable (Spearman *r* = 0.20, *p* = 0.56) (Figure 2A,B).

The distribution of patients with relevant positive reactions to 2‐HEMA, categorised by year, setting of exposure (occupational vs. non‐occupational) and sources of exposure, is shown in Table 2. An overall higher prevalence of non‐occupational (68.7%) compared to occupational cases (31.3%) was found. Non‐occupational exposures consistently showed a majority of female patients (92.4% overall). Artificial nails were the leading cause in the non‐occupational group (72.2%, 57/79), followed by dental prosthesis (12.7%, 10/79) and glues (1.3%, 1/79); other materials including paint, ECG electrode and homemade jewellery accounted for 13.9% (11/79). Artificial nails were the most frequently reported causes across both categories, comprising 72.2% of non‐occupational cases (57/79) and 75.0% of occupational cases (27/36). Dental prostheses were reported in 9.6% of all cases (11/115), glues in 6.1% (7/115), while other miscellaneous sources accounted for 11.3% (13/115). Occupational exposures showed a similar pattern, with artificial nails accounting for 75.0% of cases (27/36), while glues (16.7%, 6/36) and other materials such as paint and printing ink (5.6%, 2/36) were less frequently reported.

**TABLE 2:** Relevant positive reactions to 2‐HEMA in 115 patients: non‐occupational and occupational exposure.

Year	2019			2020			2021			2022			2023			Total		
	N. patients (%)	*M* (%)	*F* (%)	N. patients (%)	*M* (%)	*F* (%)	N. patients (%)	*M* (%)	*F* (%)	N. patients (%)	*M* (%)	*F* (%)	N. patients (%)	*M* (%)	*F* (%)	N. patients (%)	*M* (%)	*F* (%)
Non‐occupational exposure	13 (59.1)	2 (15.4)	11 (84.6)	6 (66.7)	—	6 (100)	15 (68.2)	—	15 (100)	22 (73.3)	2 (9.1)	20 (90.9)	23 (71.2)	2 (8.7)	21 (91.3)	79 (68.7)	6 (7.6)	73 (92.4)
Artificial nails	5 (38.5)	—	5 (100)	5 (83.3)	—	5 (100)	12 (80.0)	—	12 (100)	19 (86.4)	—	19 (100)	16 (69.6)	—	16 (100)	57 (72.2)	—	57 (100)
Dental prosthesis	5 (38.5)	—	5 (100)	1 (16.7)	—	1 (100)	2 (13.3)	—	2 (100)	1 (4.5)	—	1 (100)	1 (4.3)	—	1 (100)	10 (12.7)	—	10 (100)
Glues	—	—	—	—	—	—	—	—	—	1 (4.5)	1 (100)	—	—	—	—	1 (1.3)	1 (100)	—
Others*	3 (23.1)	2 (66.7)	1 (33.3)	—	—	—	1 (6.7)	—	1 (100)	1 (4.5)	1 (100)	—	6 (26.1)	2 (33.3)	4 (66.7)	11 (13.9)	5 (45.5)	6 (54.5)
Occupational exposure	9 (40.9)	—	9 (100)	3 (33.3)	2 (66.7)	1 (33.3)	7 (31.8)	—	7 (100)	8 (26.7)	—	8 (100)	9 (28.1)	3 (33.3)	6 (66.7)	36 (31.3)	5 (13.9)	31 (86.1)
Artificial nails	6 (66.7)	—	6 (100)	—	—	—	7 (100)	—	7 (100)	8 (100)	—	8 (100)	6 (66.7)	—	6 (100)	27 (75.0)	—	27 (100)
Dental prosthesis	1 (11.1)	—	1 (100)	—	—	—	—	—	—	—	—	—	—	—	—	1 (2.8)	—	1 (100)
Glues	—	—	—	3 (33.3)	2 (66.7)	1 (33.3)	—	—	—	—	—	—	3 (33.3)	3 (100)	—	6 (16.7)	5 (83.3)	1 (16.7)
Others**	2 (22.2)	—	2 (100)	—	—	—	—	—	—	—	—	—	—	—	—	2 (5.6)	—	2 (100)
Total	22 (100)	2 (9.1)	20 (90.9)	9 (100)	2 (22.2)	7 (77.8)	22 (100)	—	22 (100)	30 (100)	2 (6.7)	28 (93.3)	32 (100)	5 (15.6)	27 (84.4)	115 (100)	11 (9.6)	104 (90.4)
Artificial nails	11 (50.0)	—	11 (100)	5 (55.6)	—	5 (100)	19 (86.4)	—	19 (100)	27 (90.0)	—	27 (100)	22 (68.8)	—	22 (100)	84 (73.0)	—	84 (100)
Dental prosthesis	6 (27.3)	—	6 (100)	1 (11.1)	—	1 (100)	2 (9.1)	—	2 (100)	1 (3.3)	—	1 (100)	1 (3.1)	—	1 (100)	11 (9.6)	—	10 (100)
Glues	—	—	—	3 (60.0)	2 (66.7)	1 (33.3)	—	—	—	1 (3.3)	1 (100)	—	3 (9.4)	3 (100)	—	7 (6.1)	6 (85.7)	1 (14.3)
Others	5 (22.7)	2 (33.3)	3 (66.7)	—	—	—	1 (4.5)	—	1 (100)	1 (3.3)	1 (100)	—	6 (18.8)	2 (33.3)	4 (66.6)	13 (11.3)	5 (38.5)	8 (61.5)

* Non‐occupational others: paint (2), ECG electrodes (2), spectacle frame (1), acrylic resins for homemade jewellery (1), unknown (5).

** Occupational others: paint (1), printing ink (1).

**FIGURE 3:**
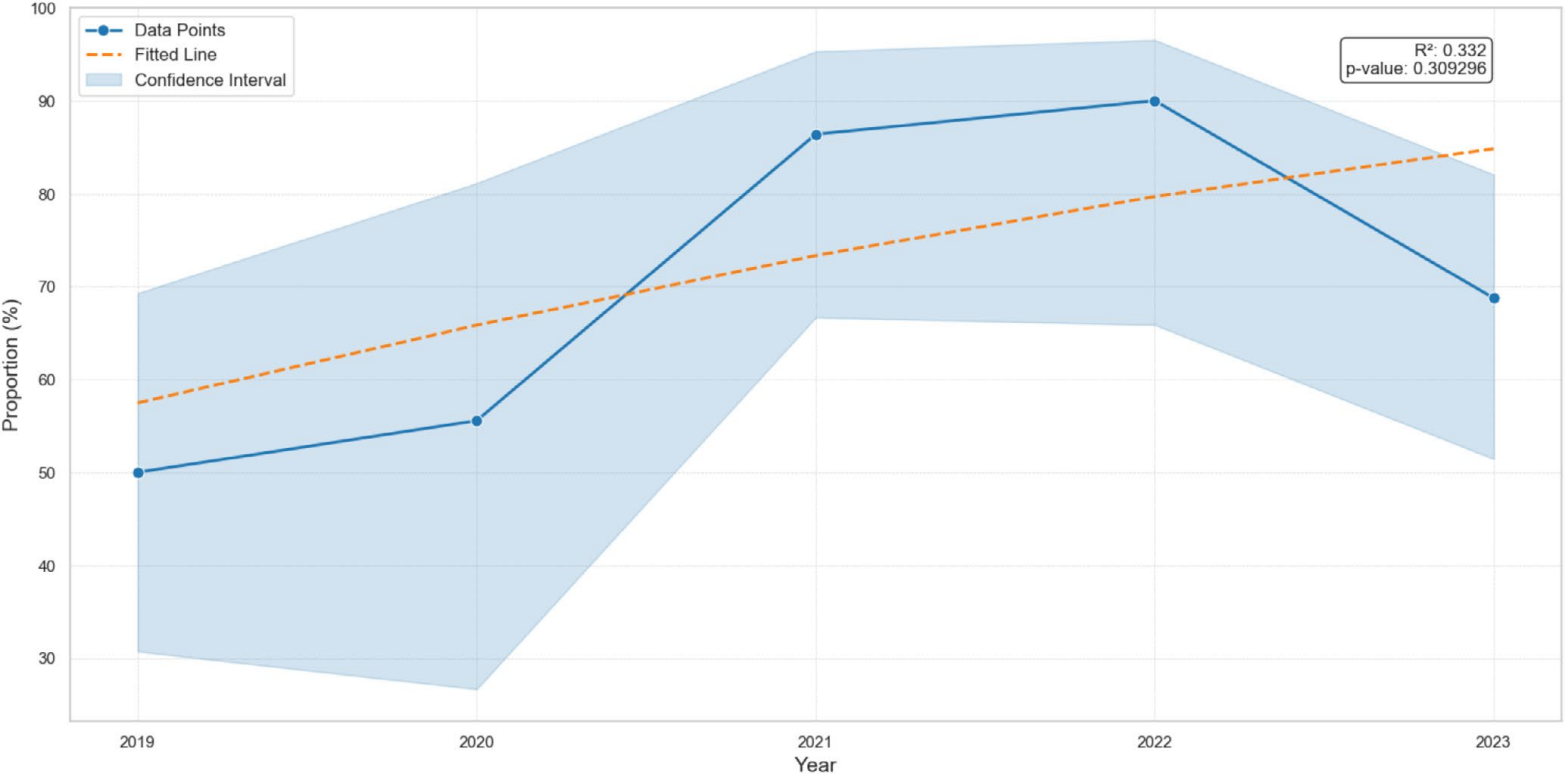
Trend of relevant positive patch test reactions to 2‐HEMA according to the proportion of patients with nail products as triggers (the confidence interval for the frequency was calculated with Wilson method is shown with the ribbon plot).

**FIGURE 4:**
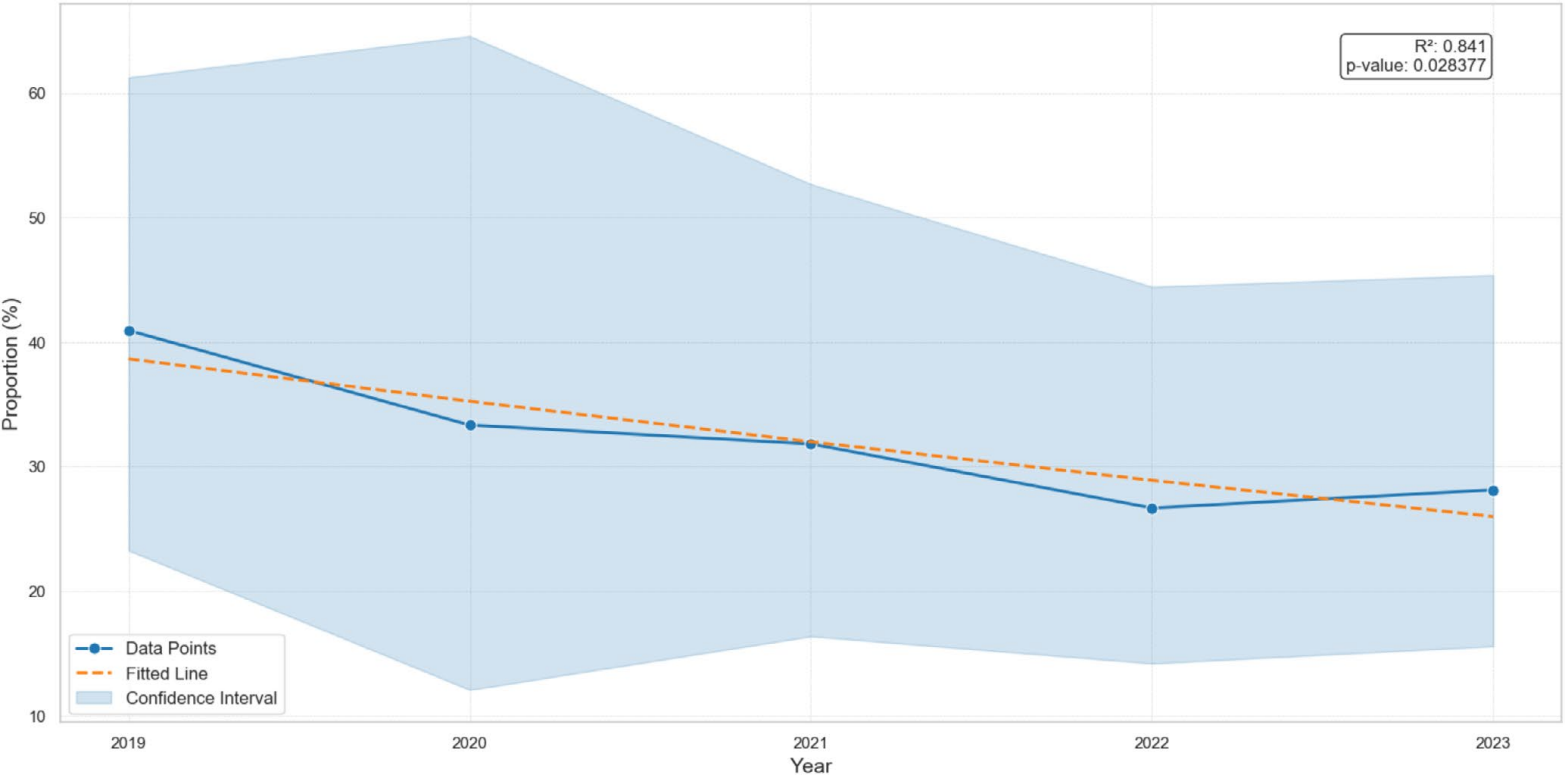
Trend of relevant positive patch test reactions to 2‐HEMA according to the proportion of patients exposed in an occupational setting (the confidence interval for the frequency was calculated with Wilson method is shown with the ribbon plot).

The proportion of patients with artificial nails as ACD trigger showed a non‐significant (*p* = 0.309) weak upward trend (Figure 3), while the proportion of patients with occupational ACD showed a statistically significant (*p* = 0.0284) downward trend (Figure 4).

The MOAHLFA index was applied to the study population using both crude proportion comparison and multiple variable logistic regression (Table 3). Considering crude comparison, all the index parameters showed statistically significant differences, apart from the “Atopy” (*p* = 0.27) and the “Face” (*p* = 0.49) variables. Accordingly, the logistic regression analysis found a statistically significant association only for the “Male” (OR 0.22, *p* < 0.001), “Occupational” (OR 4.14, *p* < 0.001), “Hand” (OR 1.99, *p* = 0.002), “Leg” (OR 0.29, *p* = 0.036) and “Age” (OR 0.54, *p* = 0.002) variables.

**TABLE 3:** MOAHLFA index in 115 patients with relevant positive reaction to 2‐HEMA.

	Subjects with relevant positive patch test reaction to 2‐HEMA (*n* = 115)	Subjects with negative patch test to 2‐HEMA (*n* = 7018)	Proportion difference *p‐value*	Crude proportion difference [95% CI]	OR *p‐value*	OR [95% CI]
Male	11 (9.6%)	2227 (31.7%)	**< 0.001**	**22.1% [16.6%–27.6%]**	**< 0.001**	**0.22 [0.12–0.41]**
Occupational	36 (31.3%)	543 (7.7%)	**< 0.001**	**23.6% [15.1%–32.1%]**	**< 0.001**	**4.14 [2.64–6.49]**
Atopy	8 (7%)	703 (10%)	0.27	3.0% [1.72%–7.71%]	0.065	0.50 [0.24–1.06]
Hand	63 (54.8%)	2217 (31.6%)	**< 0.001**	**23.2% [14.0%–32.4%]**	**0.002**	**1.99 [1.28–3.11]**
Leg	3 (2.6%)	1002 (14.3%)	**< 0.001**	**11.7% [8.68%–14.7%]**	**0.036**	**0.29 [0.091–0.92]**
Face	32 (27.8%)	1754 (25%)	0.49	2.8% [5.45%–11.05%]	0.089	1.50 [0.94–2.38]
Age > 40	48 (41.7%)	4323 (61.6%)	**< 0.001**	**19.9% [10.8%–29.0%]**	**0.002**	**0.54 [0.37–0.80]**

*Note:* Statistical significant results are highlighted in bold.

## 4 Discussion

(Meth)Acrylates are well‐established causes of ACD, with the sensitizers being the low molecular weight monomers. The present study examined the Italian 5‐year prevalence (from 2019 to 2023) of positive patch test reactions to 2‐HEMA, added to the baseline patch test series by the ESCD since 2019 [7, 13]. Amongst 7133 consecutive patch tested patients, the overall prevalence of 2‐HEMA positive reactions was 2.1% (147 cases), 1.6% (115 cases) if we consider only relevant positive patch test reactions (78.2% of all positive reactions to 2‐HEMA). Specifically, the prevalence over this 5‐year period went from 1.6% (2019) to 2.7% (2023), showing a progressively increasing trend which is consistent with European data [18, 19], albeit not statistically significant (*p* = 0.149). Our data indicate a decrease in the prevalence of 2‐HEMA allergy in 2020 (1.0%), likely attributable to the impact of the SARS‐CoV‐2 pandemic, which reduced the number of patients referred for patch testing prioritising severe cases [20]. Moreover, the 2020 lockdowns forced the Italian population to reduce social meetings, which are linked to the use of aesthetic products like nail cosmetics, the main source of exposure to acrylates in the non‐occupational setting.

In line with previous studies [5, 21], 2‐HEMA contact allergy mostly resulted in a disease concerning females, with a rate of 90.4% of relevant positive reactions (104/115 cases). Furthermore, our data showed a significant decrease in the median age of the affected women over time: from 50 years in 2019 to 28 years in 2023. In contrast, the median age of men with relevant positive patch test reactions to 2‐HEMA did not show a significant change. One of the main drivers of this difference is the increasing importance of nail products as a source of 2‐HEMA contact allergy, increasingly used by young female patients [10, 22].

Over the past 2 decades, there has been a notable change in the primary sources of exposure to (meth)acrylates, initially mainly caused by dental prostheses, glues and sealants, particularly in occupational settings [23]. Our study fully confirms the literature data demonstrating that nail products are now the most frequent cause of ACD, in both occupational (75.0%) and non‐occupational (72.2%) settings. Furthermore, we found a non‐significant upward trend in relevant positive patch test reactions to 2‐HEMA caused by artificial nails. Although dental prostheses remain a significant cause of ACD in non‐occupational settings (12.7%), their prevalence in occupational settings is consistently lower (2.8%), highlighting the effectiveness of workplace safety measures for dental professionals [24, 25].

The MOALHFA index corroborated our findings, highlighting that ACD due to 2‐HEMA predominantly affected females (male OR: 0.22, *p* < 0.001) and subjects younger than 40 years of age (age > 40 OR: 0.54, *p* = 0.002), since both the variables had an OR < 1. An additional observation involved the “occupational” variable: logistic regression analysis identified a strong association with significant positive reactions to 2‐HEMA (OR: 4.14, *p* < 0.001). However, it is important to underline that only 31.3% of 2‐HEMA‐relevant positive patch test reactions within the entire study population were linked to occupational exposure, and a significant downward trend in the proportion of patients in this group was observed over time (*p* = 0.028). The logistic regression conducted using the MOAHLFA index also showed that ACD to (meth)acrylates was strongly associated with hand eczema (OR: 1.99, *p* = 0.002) and inversely associated with leg dermatitis (OR: 0.29, *p* = 0.036). With regard to the first point, this is well known in literature and again it can be explained by the increasing role of artificial nails in (meth)acrylate contact allergy [26]. Instead, the latter depends on the lack of relevant exposure of the leg area to (meth)acrylates, sporadically found in a very small minority of patients (3 out of 115 patients, 2.6%) as previously reported [7, 27].

Several authors have documented a growing epidemic of contact allergy to 2‐HEMA depending on multiple factors [1, 19, 21, 28]. First, while the use of 2‐HEMA‐containing products is intended to be restricted to professional settings, this restriction has not been extended to non‐occupational settings. In 2018, the Scientific Commission On Consumer Safety (SCCS) released a report on the safety of 2‐HEMA in artificial nail modelling systems, concluding that 2‐HEMA is unlikely to pose a risk of sensitization due to rapid polymerisation under UV light when applied only to the nail plate [29]. Subsequently (November 2020), the use of 2‐HEMA and di‐HEMA trimethylhexyl dicarbamate (di‐HEMA TMHDC) in nail cosmetics was restricted in the context of the EU Cosmetics Regulation (EC 1223/2009), allowing only professional use [30]. The warnings “For professional use only” and “Can cause an allergic reaction” must be reported on the packaging of nail products containing 2‐HEMA, di‐HEMA TMHDC, or both [30]. From 3 September 2021, products containing these substances and not complying with those conditions should no longer have been made available on the Union market.

Nevertheless, these products remain widely available, particularly through online marketplaces, which can be easily accessed even by very young customers. The purchase of home kits for nail cosmetics has notably increased since the SARS‐CoV2 pandemic [28, 31]. Additionally, while the Registration, Evaluation, Authorisation and Restriction of Chemicals (REACH) regulations primarily govern the use of 2‐HEMA and di‐HEMA TMHDC [29], acrylate‐containing products can release a variety of allergenic compounds, such as hydroxypropyl methacrylate, isobornyl methacrylate and trimethylolpropane triacrylate [10]. Compounds such as these can be either present as impurities or formed because of the inappropriate domestic use of UV‐lamps, making effective monitoring challenging, especially for domestic or small‐scale industrial use [32, 33].

The other key factor contributing to the rise in 2‐HEMA contact allergy is the increased use of nail products for cosmetic purposes [34, 35, 36], particularly prominent amongst younger girls, heavily influenced by the prevailing trends on social media [28]. Several authors are indeed more frequently reporting case reports of severe ACD in paediatric patients [37, 38], which can be severely impactful on their quality of life. In fact, contact allergy to (meth)acrylates can influence future job choices (e.g., dentistry [39], beauty industry [22], manufacturing [1]), requiring strict avoidance of these compounds. Also, in patients allergic to 2‐HEMA caution should be used towards medical devices, such as orthopaedic or dental prosthesis, even if not necessarily an acrylate implant causes local or systemic reactions [40]. Caution should also be used towards electrodes for electrocardiogram [41, 42] or transcutaneous electrical nerve stimulation [43], surgical [44] and medical glues [45], diathermic pads [46], hearing aids [47], headphones [48], vape pens [49], and eyeglass frames [50], which have all been reported to be causes of acrylates‐related ACD.

In conclusion, the findings of this study highlight the growing prevalence of ACD to 2‐HEMA in Italy, primarily driven by the increasing use of nail products, particularly amongst young women and secondly by the failure of 2‐HEMA regulation. In fact, the current regulatory measures under European legislation aim to restrict the use of 2‐HEMA‐containing products to professional settings, but do not consider that several other acrylates cross‐reacting with 2‐HEMA are frequently contained in nail cosmetics [19], still easily accessible to the general public, mainly through online platforms. The emerging exposure source represented by nail products highlights the need for more stringent enforcement of regulations and public awareness campaigns regarding the associated risks. Moreover, this study calls for further multicentre European research, beyond the study recently performed by Wilkinson et al. [11], to confirm our findings and to develop more effective preventive strategies and adequate legislation. Enhanced monitoring and regulation, coupled with public education, could play a pivotal role in mitigating this emerging public health issue.

## Author Contributions

**Elena Sofia Caroppo:** conceptualization, methodology, writing – original draft, investigation, data curation, formal analysis. **Gabriele Casciola:** conceptualization, methodology, data curation, investigation, writing – original draft, formal analysis. **Katharina Hansel:** conceptualization, methodology, writing – review and editing, supervision, visualization. **Alba Guglielmo:** investigation, visualization. **Silvia Mariel Ferrucci:** investigation, visualization. **Fabrizio Guarneri:** investigation, visualization, software, data curation. **Maria Michela Lauriola:** investigation, visualization. **Maddalena Napolitano:** investigation, visualization. **Cataldo Patruno:** investigation, visualization. **Paolo Romita:** investigation, visualization. **Donatella Schena:** investigation, visualization. **Ilaria Trave:** investigation, visualization. **Leonardo Bianchi:** conceptualization, methodology, investigation, writing – review and editing. **Luca Stingeni:** conceptualization, methodology, data curation, validation, visualization, writing – review and editing, supervision. **Elisa Marzola**: investigation. **Francesco Bellinato:** investigation. **Paolo Calzari:** investigation. **Francesca di Vico:** investigation. **Rosella Gallo:** investigation. **Roberta Giuffrida:** investigation. **Benedetta Tirone:** investigation. **Marta Tramontana:** investigation.

## Conflicts of Interest

The authors declare no conflicts of interest.

## Data Availability

The data that support the findings of this study are available from the corresponding author upon reasonable request.

## Appendix A

2‐HEMA Study Group: Elisa Marzola (Section of Dermatology and Infectious Diseases, Department of Medical Sciences, University of Ferrara, Ferrara, Italy); Francesco Bellinato (Section of Dermatology and Venereology, Department of Medicine, University of Verona, Verona, Italy); Paolo Calzari (Dermatology Unit, Fondazione IRCCS Ca' Granda Ospedale Maggiore Policlinico, Milan, Italy); Francesca di Vico (Section of Dermatology, Department of Clinical Medicine and Surgery, University of Naples Federico II, Naples, Italy); Rosella Gallo (Section of Dermatology, Department of Health Sciences, University of Genoa, IRCCS, Ospedale Policlinico San Martino, Genoa, Italy); Roberta Giuffrida (Section of Dermatology, Department of Clinical and Experimental Medicine, University of Messina, Messina, Italy); Benedetta Tirone (Section of Dermatology, Department of Precision and Regenerative Medicine and Jonian Area, University Aldo Moro of Bari, Bari, Italy); Marta Tramontana (Dermatology Section, Department of Medicine and Surgery, University of Perugia, Perugia, Italy).
